# Supplementary material for: Role of the volume conductor on simulations of local field potential recordings from deep brain stimulation electrodes
Source: PLoS One. 2023 Nov 27;18(11):e0294512. doi: 10.1371/journal.pone.0294512 (PMC10681243; doi:10.1371/journal.pone.0294512)
Supplement: S1 File — (PDF) [file pone.0294512.s001.pdf]

Supporting information

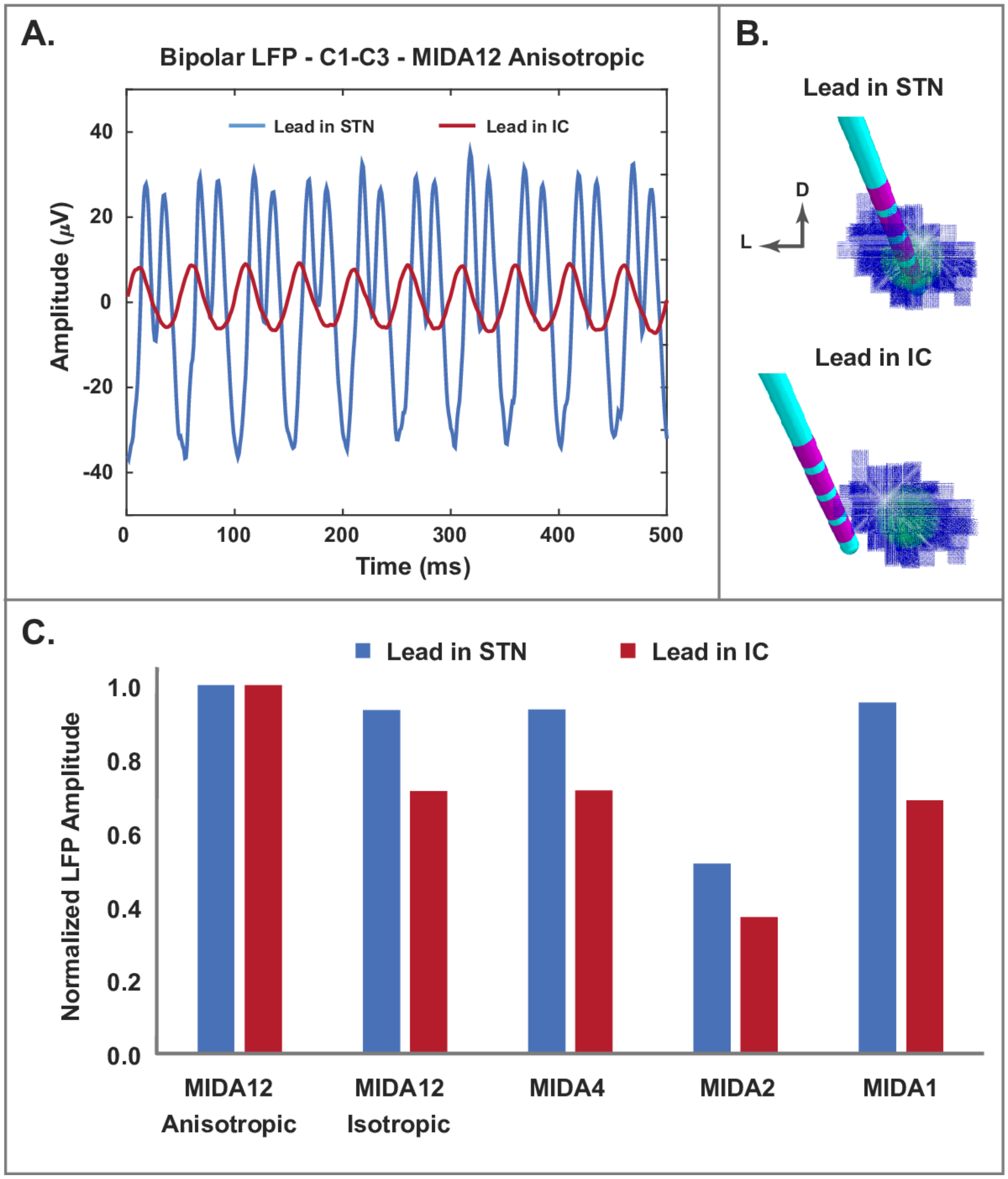

S1 Fig: Effect of volume conductor complexity on simulated LFPs when the DBS lead was placed in the Subthalamic Nucleus (STN) and in the Internal Capsule (IC). A) Bipolar LFP between contact 1 and 3 simulated with MIDA12 anisotropic volume conductor model. B) Coronal view depicting DBS lead in the STN and in the IC. C) Comparison of normalized LFP amplitude simulated using all five VC variants when the DBS lead was placed in the STN and in the IC. LFP amplitudes using different VC model variants were normalized with the amplitude of LFP simulated with MIDA12 anisotropic VC model.

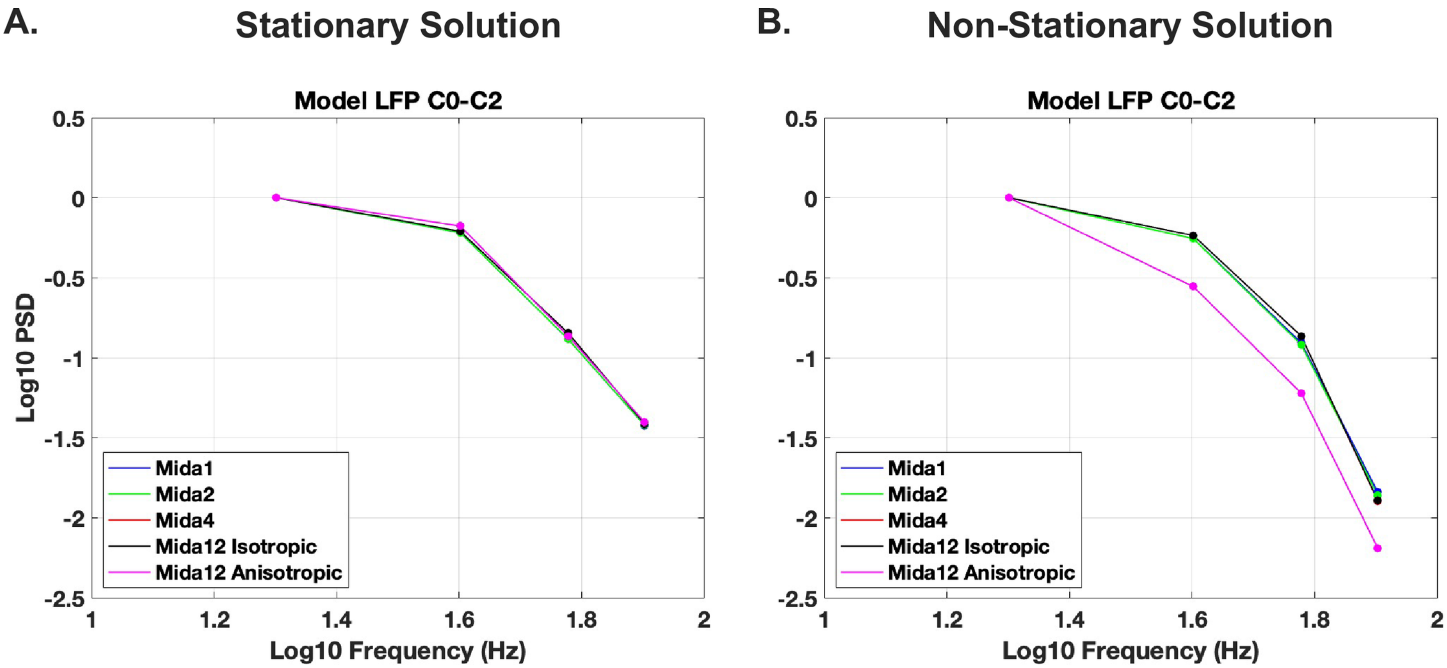

S2 Fig: Effect of Volume Conductor Heterogeneity. Log-Log plot of LFP Power Spectral Density (PSD) simulated with various VC models using a (A) Stationary solution (B) Non-stationary solution.

S1 Table: Neuronal densities for each sector of the STN

STN Sector	Neuronal Density, neurons / mm^3
Anterior dorsal	1207
Anterior central	1148
Anterior ventral	1196
Middle dorsal	1431
Middle central	1472
Middle ventral	1611
Posterior dorsal	1269
Posterior central	1626
Posterior ventral	1872

S2 Table: Ion Channel Conductances

Channel Conductances	Soma (S/cm^2)	Dendrites (S/cm^2)
Na	1.11e-2	6.5e-8
NaP or NaL	0.83e-5	5.26e-6
KDR	3.84e-3	3.84e-3
Kv31	1.61e-2	1.5e-2
sKCa	1.23e-4	1.00e-4
HCN	1.01e-3	1.00e-3
CaT	0.00	1.00e-3
CaN	1.15e-3	1.20e-2
CaL	9.50e-4	2.00e-3

Jitter added to the synchronous neurons

Scipy function *truncnorm.rvs* (with ±2 bounds) was used to generate jitter from a truncated normal distribution. This jitter when added to the spike times give rise to synchronous neuron synaptic input timing.

Jitter added to the asynchronous neurons

Numpy function *random.exponential()* was used to generate jitter from an exponential distribution. This jitter when added to the spike times give rise to asynchronous neuron synaptic input timing.
