# Supplementary material for: Role of the volume conductor on simulations of local field potential recordings from deep brain stimulation electrodes
Source: PLoS One. 2023 Nov 27;18(11):e0294512. doi: 10.1371/journal.pone.0294512 (PMC10681243; doi:10.1371/journal.pone.0294512)
Supplement: Attachment — Submitted filename: Noor VC response.docx [file pone.0294512.s002.docx]

**PONE-D-22-10944**: Role of the Volume Conductor on Simulations of Local Field Potential Recordings from Deep Brain Stimulation Electrodes

**Response to Reviewer Reports**

**Reviewer #1**: This paper deals with exploring a DBS Local field potential model and analyses its complexity. The work is sound and publishable. The following are the minor comments:

(1) In Introduction, contribution of the study should be provided.

Response: We added two sentences to the end of the Introduction to provide a summary of our findings.

(2) In Introduction, some comparison statements with the existing works and complexity in achieving results should be mentioned.

Response: We added two sentences to the middle of the Introduction to highlight previous work addressing the effect of VC model complexity on the simulation of EEG signals. We are unaware of previous VC modeling studies that have explicitly addressed the topics of this study with reference to LFP recordings.

(3) In general, the method section is brief. Three models are presented. Is it possible to describe a method of combining these three models. This will provide a combined approach used as a method to the readers for clarity.

Response: We agree with the Reviewer that our description of the modeling methods is brief. However, those technical details are already documented several times in the predecessor papers, specifically Lempka & McIntyre [2013] and Maling et al. [2018]. Therefore, in the first paragraph of Methods we have added a sentence referring to the approach we used to combine model source with the volume conductor.

(4) What are furture research directions.

Response: We added a new paragraph at the end of the Discussion that describes our future research directions with the series of DBS LFP modeling papers.

**Reviewer #2**: Thank you for this work. While the paper is well-written and the results are clear, I had some comments and questions below which prevent immediate recommendation for publication. Kindly respond to the review and resubmit. The comments are in a separate attachment.

There is increasing interest in local field potentials as a decoding signal for Deep Brian Stimulation (DBS). Motivated by this interest, the lab of the senior investigator has published several papers previously modeling the local field potential in the vicinity of a DBS electrode implanted in the subthalamic nucleus (STN), the most common target of DBS for Parkinson’s Disease.

The models comprise of many thousands of compartmental models of STN cells placed in a volume conductor. The neuronal morphology in these models is adopted from experimental reconstructions of STN neurons in macaque and synaptic connections also derived from experiments. Thousands of these model neurons are placed within a volume conductor (VC) in the vicinity of the DBS electrode. Driving these neurons by synaptic inputs, either correlated or uncorrelated, and examining the voltage recorded at the DBS electrode contact because of the transmembrane currents induced in the neurons by the synaptic drive is the crux of their LFP modeling method. In previous work, the VC has been homogenous and isotropic. In this paper the authors ask whether inhomogeneity and anisotropy of the VC significantly affects the results. They find that while model complexity does affect the results, the effect is relatively small and could be incorporated by suitably changing the parameters of the simple model.

While the paper is well-written and the results seem reasonable in terms of accuracy, I had several comments/questions, some minor, others more important, that prevent me from recommending publication. It would be great of the authors address these points in the revision. These are below:

1) Why has the number of neurons changed from Soheil et al, 2021 to this submission?

Response: The STN model used in Noor & McIntyre [2021], and Maling et al. [2018], was defined from patient-specific imaging data on a 52-year old male with Parkinson’s disease. The STN model used in this submission is based on the MIDA head model, which is derived from the imaging data of a 29-year-old healthy female volunteer [Iacono et al., 2015]. These two different brains have subtle differences in the volume and shape of their STN. Therefore, the exact number of STN neuron models that populate the nucleus is slightly different. Nonetheless, both examples have a neural density that is consistent with histological estimates for the human STN.

2) In Maling et al, the authors suggest that absence of a more complex VC is responsible for the inability of the model to reproduce the 1/f tail observed in the experimental LFPs. But this paper does not examine the 1/f behavior. Could the authors comment?

Response: In Maling 2018, we proposed that the homogeneity of synaptic inputs and absence of ionic diffusion were responsible for the absence of 1/*f behavior*. However, Bédard and Destexhe 2004 suggested that volume conductor heterogeneity could contribute to *1/f* scaling in the power spectral density plot. To investigate this further, we performed simulations in the frequency domain using “Fourier FEM”. We found that tissue heterogeneity had relatively modest effects on the *1/f* scaling (see supplemental figure) leading us to believe that *1/f* behavior is primarily dictated by ionic diffusion rather than tissue heterogeneity. This conclusion is supported by recent studies [Halnes 2016, Bédard 2022]. We have added a new paragraph to the end of the Discussion to address these points.

3) One of the major features that can influence the LFP is the geometric arrangement of the neurons. As the authors well know, projection neurons in the STN project to many different structures which should result in different geometries (Sato et al, JCN, 2000). But as far as I can tell, the model has neurons oriented in such a way as to accentuate the LFPs. Can the authors comment on what it would take to make the model more realistic along these lines and how this would affect the results? In terms of number of neurons their simulations appear to at close to human scale (Bokulic et al, Frontiers in Neuroanatomy, 2021).

Response: Yes, the DBS LFP model consists of a STN neuron density that aligns with human histological estimates. While we acknowledge that neuron orientation is generally an important factor in LFP simulation, previous studies have reported that STN neurons are oriented parallel to the long axis of the nucleus [Hammond 1983, Yelnik 1979]. Neurons’ orientation in our model is consistent with this observation. Moreover, our paper primarily focuses on exploring the impact of volume conductor heterogeneity on LFPs, and the differences in electric current due to neural orientation would not affect our findings.

4) Finally, Lempka et al, 2013 from this group argued that since the FEM model is linear, changing the number of neurons would change the modeled LFP linearly. But doesn’t this raise the question of why one needs multiple neurons at all? Perhaps fewer neurons, placed appropriately relative to the DBS lead could lead to the same results and would allow exploration of aspects such as heterogeneity of dendritic orientations? Could the authors comment on this issue?

Response: Yes, we agree with the Reviewer, and this general strategy represents our goal moving forward with DBS LFP models. The basic modeling philosophy of our group is to start with a highly detailed model representation, match that detailed model up with known anatomy and physiology, and then reduce the model down to a simpler representation that can still capture most of the features of the detailed model. This manuscript is focused on understanding the impact of using simplified VC models. Our next manuscript will be focused on understanding the impact of using simplified neuron/source models. Along that line, our recent results suggest that consolidating our ~200K STN neuron models (with ~ 8 million individual sources) into a collection of ~1000 anatomically positioned STN sources, can mimic the detailed model with a high degree of fidelity. This simplified model will provide a much faster tool to perform patient-specific inverse-model beta-source localizations in the STN. We added a new paragraph at the end of the Discussion that describes this future research direction.
